# Identification of anoikis-related biomarkers linking immune infiltration to acute myocardial infarction

**DOI:** 10.1590/1414-431X2025e14989

**Published:** 2026-01-09

**Authors:** Yang Zhao, Shangfen Wang, Huiying Ji, Lihui Han, Shiai Wang, Xunli Yin, Qian Zhao

**Affiliations:** 1 Department of Cardiology, The Second Affiliated Hospital of Shandong First Medical University, Tai'an, Shandong Province, China; 2 Department of Cardiology, The Seventh People's Hospital of Jinan, Jinan, Shandong Province, China; 3 Department of Cardiovascular Medicine, The Seventh People's Hospital of Jinan, Jinan, Shandong Province, China; 4 Department of Cardiovascular Medicine, Central Hospital Affiliated to Shandong First Medical University, Jinan Central Hospital, Jinan, Shandong Province, China

**Keywords:** Acute myocardial infarction, Anoikis, Immune infiltration, Biomarkers, Nomogram

## Abstract

This study was designed to identify new biomarkers for early diagnosis of acute myocardial infarction (AMI). GSE66360 and GSE48060 datasets were downloaded from the Gene Expression Omnibus (GEO) database. The “limma” tool was used to screen differentially expressed genes (DEGs). A total of 557 anoikis-related genes (ARGs) were obtained from the GeneCard database. Differentially expressed ARGs (DEARGs) were obtained by intersecting DEGs with ARGs. The least absolute shrinkage and selection operator (LASSO), support vector machine (SVM), and Random Forest (RF) were used to screen the hub DEARGs. Real-time quantitative polymerase chain reaction (RT-qPCR) was used to determine the expression of hub DEARGs. A total of 21 DEARGs were obtained, all of which were up-regulated in AMI samples. Functional enrichment analysis showed that the DEARGs were mainly enriched in peptidase activity and extracellular matrix. Immune cell infiltration analysis revealed a significant difference in 14 immune cells between the AMI and normal groups. Nine feature risk genes, including *ITPRIP*, *MMP9*, *NAMPT*, *CDKN1A*, *PLAUR*, *PLAU*, *SERPINA1*, *THBS1*, and *FN1* were screened by LASSO, SVM, and RF. The RT-qPCR analyses verified that the feature genes were up-regulated in AMI patients, which were basically consistent with the main bioinformatics analysis results. We also validated 9 hub DEARGs in the GSE48060 dataset and constructed a nomogram by integrating these DEARGs. This study analyzed the differential expression of ARGs and immune profiles in AMI and normal samples, screened 9 risk feature genes for predicting AMI, and provided a theoretical basis for the immunotherapy regimen of AMI.

## Introduction

Acute myocardial infarction (AMI) is myocardial necrosis resulting from the interruption of blood flow following the rupture of unstable plaques in the coronary artery (1). Approximately 75% of patients diagnosed with AMI subsequently develop heart failure, which represents a significant cause of mortality on a global scale (2). The cornerstone of current treatment for AMI is the rapid and complete reperfusion of occluded coronary arteries. Nevertheless, a considerable number of patients experience unfavorable clinical outcomes due to insufficient timely myocardial reperfusion (3). The early diagnosis and interventional treatment of AMI can significantly reduce myocardial injury, improve prognosis, and decrease mortality (4).

The combination of biomarker detection and electrocardiogram (ECG) analysis is the main strategy for the diagnosis of AMI. Currently, multiple biomarkers of cardiac injury have been used to diagnose AMI, such as aspartate transaminase (AST), creatine kinase (CK), lactate dehydrogenase (LDH), and cardiac troponins (cTns) (5). However, these biomarkers still lack specificity for the diagnosis of AMI. Despite a recent study indicating that nanomaterials can enhance the accuracy of various cardiac biomarkers, the number of approved clinical trials for AMI remains limited (6). Moreover, multi-biomarker methods can provide more information for the diagnosis and treatment of AMI. Therefore, it is necessary to find new biomarkers for early diagnosis of AMI, thereby improving prognosis and mortality.

Anoikis is a form of cell apoptosis that occurs during the cell's detachment from the extracellular matrix (ECM), disrupting integrin ligation (7). It is a critical mechanism for preventing dysplastic cell growth and inappropriate matrix attachment, thereby regulating the occurrence of metastatic cancer, cardiovascular disease, and diabetes (8). The cardiac ECM provides structural tissue for the myocardium, and the imbalances in ECM production and degradation are associated with adverse effects on cardiac remodeling and dysfunction (9). It is speculated that the loss of ECM following myocardial infarction may trigger cell apoptosis via the anoikis mechanism (10). However, the mechanism of anoikis in the pathogenesis of AMI has been rarely explored.

Evidence suggests that anoikis regulates immune responses and plays a crucial role in AMI occurrence (11). In AMI patients, the onset of acute myocardial ischemia can induce an initial pro-inflammatory response. Subsequently, it enters the anti-inflammatory reparative phase through the fine and complex interaction between the heart itself and the components of the immune response (including neutrophils, monocytes, macrophages, dendritic cells, and lymphocytes) (12). It is speculated that persistent or chronic inflammation after AMI may lead to adverse left ventricular remodeling and lead to heart failure (13). The immune infiltration analysis can provide additional diagnostic and therapeutic targets for preventing heart failure after myocardial infarction. Therefore, an in-depth study of the different immune characteristics between normal and AMI samples may help elucidate the changes in anoikis and related genes, providing new insights for the treatment of AMI.

This study was the first to comprehensively analyze the differential expression of anoikis-related genes (ARGs) and immune profiles in normal and AMI peripheral blood samples. A machine learning algorithm was used to screen hub genes and build a nomogram model for the early diagnosis of AMI.

## Material and Methods

### Data collection and preparation

Two datasets of gene expression profiles from AMI-related peripheral blood samples, GSE66360 (https://www.ncbi.nlm.nih.gov/gds/?term=GSE66360) and GSE48060 (https://www.ncbi.nlm.nih.gov/gds/?term=GSE48060), were downloaded from Gene Expression Omnibus (GEO) database. The GSE66360 was used as a training dataset, and the GSE48060 was used as a validation dataset. The R packages “GEOquery” and “AnnoProbe” were used to download the gene expression matrix, and the GPL570 platform was used to annotate the probe onto the gene. Under the condition of a correlation coefficient >0.4, a total of 557 anoikis-related genes (ARGs) were obtained from GeneCard database (https://www.genecards.org/).

### Analysis of differentially expressed genes

After the preparation of the GSE66360 dataset, data normalization was performed using the normalizeBetweenArrays function, followed by screening for differentially expressed genes (DEGs) with the “limma” package. Adjusted P-values <0.05 and |log_2_Foldchange (FC)| >1 were considered significant. The volcano plot and heat map of DEGs were drawn using R packages “ggplot” and “pheatmap”.

### Identification of DEARGs and functional enrichment analysis

Differentially expressed ARG (DEARGs) were obtained by intersecting DEGs with ARGs. Next, we performed the functional enrichment analysis using the R package “clusterProfiler”, including Gene Ontology (GO) and Kyoto Encyclopedia of Genes and Genomes (KEGG) enrichment analysis. The Benjamini-Hochberg method was used to adjust the P-value, and the screening criterion was a corrected P-value <0.05.

### Immune cell infiltration analysis

To determine the differences between different clusters and the immune cell infiltration between the AMI group and normal group, CIBERSORT (https://cibersortx.stanford.edu) was used to evaluate the proportion of immune cells in each sample based on the whole transcriptome data. The specific parameters used were permutations=100 and quantile normalization=TRUE. The Wilcoxon signed-rank test was used to calculate the difference in cell proportion between groups. The R package “ggcorrplot” was used to calculate the correlation between immune cells. The Pearson correlation coefficient between DEARGs and immune cells was calculated, and the results are reported in the form of a heatmap using the R package “pheatmap”.

### Identification and verification of hub DEARGs in AMI

The “glmnet” was used for the Least Absolute Shrinkage and Selection Operator (LASSO) to select hub DEARGs according to the minimum lambda value. The R package “e1071” was used for Support Vector Machine (SVM) to screen hub DEARGs, and 10-fold cross-validation was used to validate. Based on the selected diagnostic signs, a diagnostic model was created using SVM and Random Forest (RF). We then evaluated the predictive accuracy of the training dataset and validation dataset by drawing receiver operating characteristic (ROC) curves using the R package “pROC”.

### Real-time quantitative polymerase chain reaction (RT-qPCR) of hub DEARGs

To further validate the expression of hub DEARGs in clinical samples, this study collected 17 human blood samples from 8 AMI patients and 9 healthy controls. All AMI patients were recruited from the Department of Cardiology of the Second Affiliated Hospital of Shandong First Medical University and met the 2017 European Society of Cardiology (ESC) ST-segment elevation myocardial infarction (STEMI) criteria (14). The healthy controls matched for gender and age with the AMI patients were selected from the Health Examination Center as controls. Comorbidities, such as hypertension and diabetes, showed no significant differences between the two groups. The detailed clinical information of the subjects is presented in Supplementary Table S1. Peripheral blood samples (5 mL) were collected in the morning after an overnight fast. This study was conducted in accordance with the principles of the Helsinki Declaration and was approved by the Ethics Committee of the Second Affiliated Hospital of Shandong First Medical University (2023-H-172). This experiment obtained informed consent for the use and publication of clinical data from each patient and their family members.

The blood RNA extraction kit (Magen, R4163-02, China) was employed to extract total RNA from peripheral blood. The FastQuant cDNA First Strand Synthesis Kit (TIANGEN, KR106, China) was used for the reverse transcription of cDNA. The fluorescence quantitative PCR instrument (Gene-9660, BIOER, Hangzhou, China), SuperReal PreMix Plus (SYBR Green, China), and SuperReal fluorescence quantitative premix enhanced version (FP205, TIANGEN, China) were employed for RT-qPCR to examine gene expression. Samples were run on triplicate plates. GADPH and ACTB served as controls. The data were obtained by the 2^-ΔΔCT^ method. The primers used are listed in Supplementary Table S2.

### Consensus clustering analysis

To identify various patterns of anoikis in AMI, the R package “Consens-usClusterPlus” was used to perform unsupervised cluster analysis of DEARGs. The optimal subtype number (*k*) was determined using the cumulative distribution function (CDF) curve and consensus score, and we performed principal component analysis (PCA) using R package “ggplot2” to determine if the classification is applicable. Given the known progression from AMI to heart failure, we validated the clinical relevance of anoikis-related AMI subtypes by performing a consensus clustering analysis on the GSE59867 dataset, which covered prognostic information of heart failure.

### Establishment and validation of a nomogram

A nomogram was constructed based on the selected hub DEARGs. The discriminative power of the nomogram was evaluated by the area under the curve (AUC) created through the rms package. We evaluated the degree of consistency between expected probability and actual probability through calibration curves. The decision curve analysis (DCA) was used to evaluate the clinical utility and advantages of the nomogram.

### Statistical analysis

GraphPad Prism software version 8.0 (USA) was used for statistical analyses. The means of the groups were compared using Student's *t*-test. A P-value <0.05 was considered statistically significant.

## Results

### Identification of DEARGs in AMI

**Figure 1:**
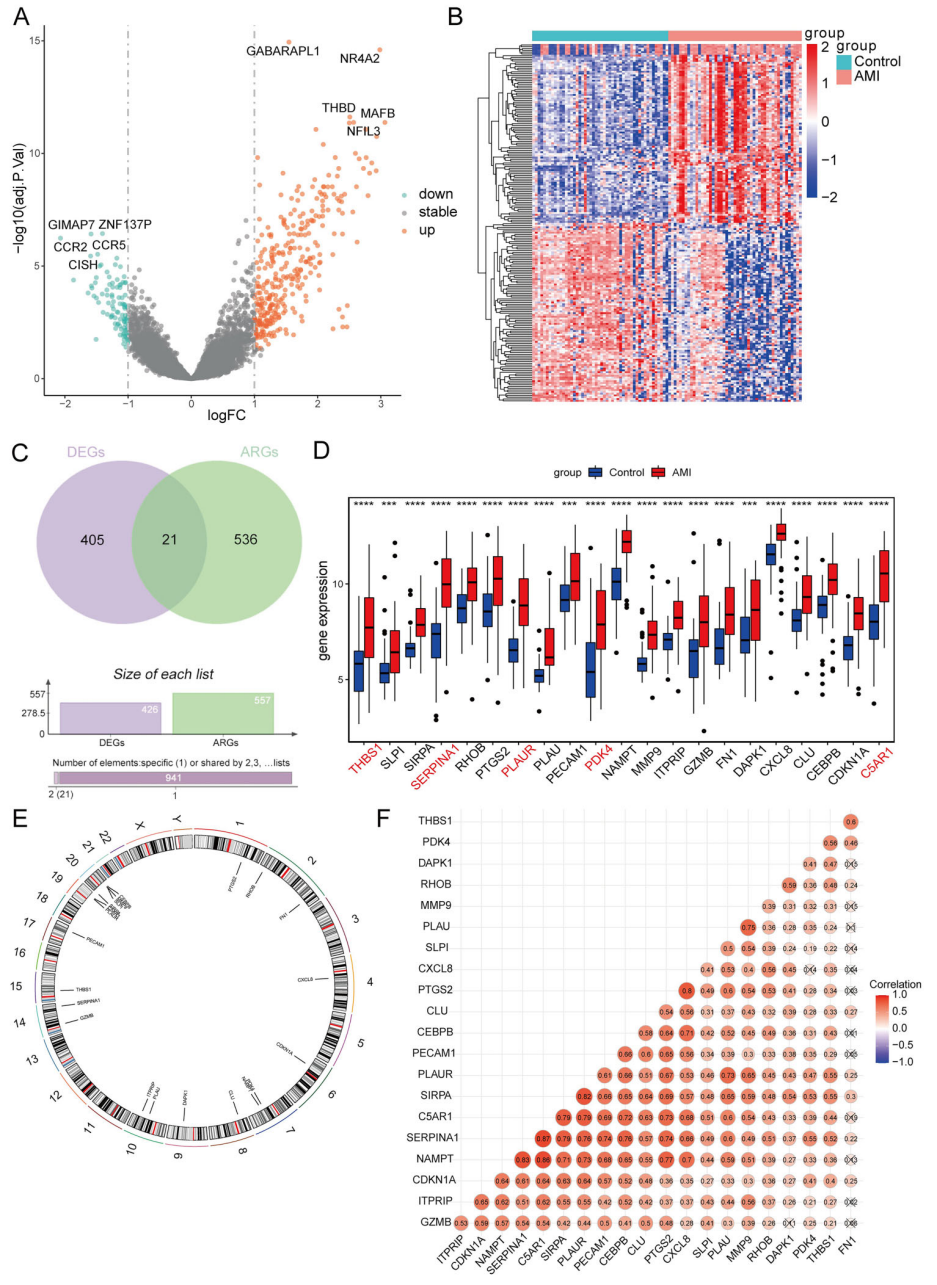
Identification of differentially expressed genes (DEGs) and differentially expressed anoikis-related genes (DEARGs). **A**, Volcano plot of DEGs, where blue dots indicate down-regulated genes, gray dots indicate stable genes, and red dots indicate up-regulated genes. **B**, Heat map of DEGs, where each column corresponds to the expression profile of an acute myocardial infarction (AMI) sample, and each row corresponds to a gene. **C**, Venn diagram of DEGs and ARGs. **D**, Expression of 21 DEARGs in normal and AMI samples. The top 5 genes with the most significant differential expression are marked in red text (sorted in descending order by |log_2_FC|). The blue group represents normal samples, while the red group represents AMI samples. Data are reported as median and interquartile range. ***P<0.001, ****P<0.0001 (Kruskal-Wallis test). **E**, Relative positions of the 21 DEARGs on the chromosomes. **F**, Correlation heat map of the 21 DEARGs, where red represents positive correlation, while blue represents negative correlation.

After data normalization, the expression distribution was homogenized across samples (Supplementary Figure S1). A total of 426 DEGs were identified in AMI samples under the cutoff value of adjusted P-value <0.05 and |log_2_FC| >1. The volcano and heat maps of DEGs are shown in Figure 1A and B, with 328 up-regulated genes and 98 down-regulated genes. A total of 21 DEARGs were obtained by intersecting DEGs and ARGs, all of which were up-regulated in AMI samples (Figure 1C and D). Among them, the top 5 genes with the most significant differential expression were *SERPINA1*, *C5AR1*, *PLAUR*, *PDK4*, and *THBS1*. The chromosome locations of 21 DEARGs are shown in Figure 1E. The correlation analysis of 21 DEARGs showed that these genes exhibited strong synergistic effects (Figure 1F).

### Functional enrichment analysis of DEARGs

**Figure 2:**
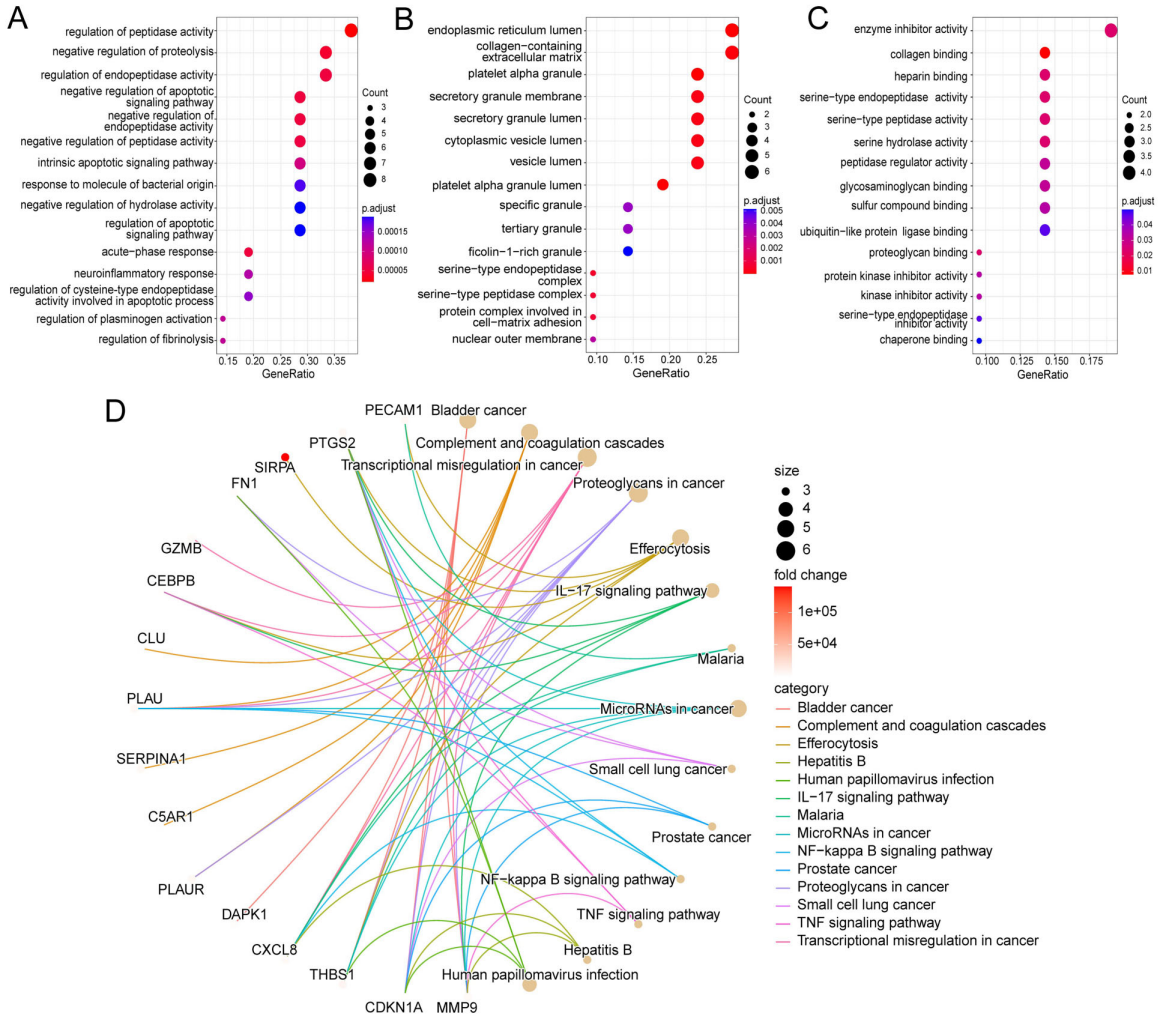
Functional enrichment analysis of differentially expressed anoikis-related genes (DEARGs). **A**, Biological process of GO enrichment analysis. **B**, Cell components of GO enrichment analysis. **C**, Molecular function of GO enrichment analysis. **D**, KEGG pathway of DEGs. GO: Gene Ontology; KEGG: Kyoto Encyclopedia of Genes and Genomes.

The GO functional enrichment analysis indicated that DEARGs were primarily associated with regulating enzyme activity in biological processes (BP), including “regulation of peptidase activity”, “negative regulation of proteolysis”, “regulation of endopeptidase activity”, and other terms (Figure 2A). The DEARGs were mainly enriched in cellular components such as “endoplasmic reticulum lumen”, “collagen-containing extracellular matrix”, and “platelet alpha granule” (Figure 2B). For molecular functions, the main terms of enrichment were “enzyme inhibitor activity”, “collagen binding”, and “heparin binding” (Figure 2C). In addition, the KEGG pathway of DEARGs was found to be mainly enriched in “Bladder cancer”, “Complement and coagulation cascades”, “Efferocytosis”, “Hepatitis B”, “Human papillomavirus infection”, and “IL-17 signaling pathway” (Figure 2D).

### Immune cell infiltration analysis

**Figure 3:**
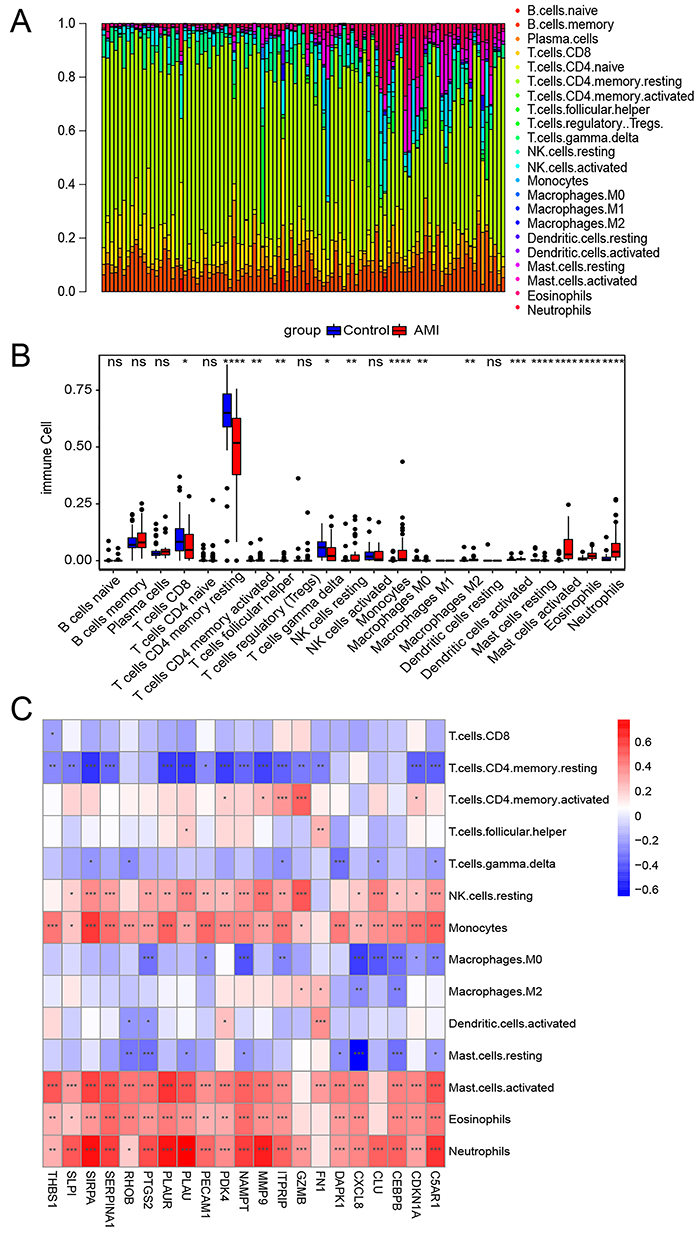
Immune infiltration analysis. **A**, Percentage of immune cells. **B**, Immune cell difference between normal and acute myocardial infarction (AMI) samples. **C**, Correlation between 21 differentially expressed anoikis-related genes (DEARGs) and 14 differential immune cells. Data are reported as median and interquartile range. *P<0.05, **P<0.01, ***P<0.001, ****P<0.0001 (Kruskal-Wallis test). ns: non-significant.

To identify the immune cell characteristics of AMI, we analyzed the proportion of 22 types of immune cells in each sample in the GSE66360 dataset (Figure 3A). Compared to the control group, the infiltration levels of T cells CD8, resting memory CD4 T cells, T cells gamma delta, M0 macrophages, and resting mast cells in the AMI group were lower, while those of CD4 memory activated T cells, follicular helper T cells, resting NK cells, monocytes, M2 macrophages, activated dendritic cells, activated mast cells, eosinophils, and neutrophils were higher (Figure 3B). Moreover, correlation analysis showed that DEARGs were positively correlated with the infiltration level of resting NK cells, monocytes, activated mast cells, eosinophils, and neutrophils, and negatively correlated with resting memory CD4 T cells and M0 macrophages (Figure 3C).

### Identification of the hub feature genes

**Figure 4:**
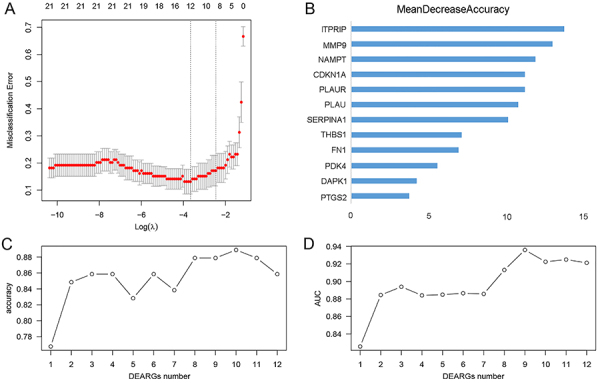
Identification of hub feature genes by LASSO and SVM. **A**, Feature genes identified by LASSO. **B**, Random Forest sorting results of hub feature genes. **C** and **D**, 10-fold cross-validation was used to calculate AUC, and the optimal parameter was determined to be 9. LASSO: least absolute shrinkage and selection operator; SVM: support vector machine; AUC: area under the ROC curve.

Among 21 DEARGs, 12 genes associated with the occurrence of AMI were screened by LASSO. According to the order of RF sorting results, *ITPRIP*, *MMP9*, *NAMPT*, *CDKN1A*, *PLAUR*, *PLAU*, *SERPINA1*, *THBS1*, *FN1*, *PDK4*, *DAPK1*, and *PTGS2* were obtained (Figure 4A and B). Then, 10-fold cross-validation was used to calculate the accuracy of the AUC. As illustrated in Figure 4C and D, upon reaching the ninth mRNA, the AUC value undergoes a transition from an increasing to a decreasing pattern, ultimately attaining its maximum value. Consequently, the top 9 DEARGs were identified as the optimal biomarkers.

### Validation of the hub DEARGs by ROC curve

**Figure 5:**
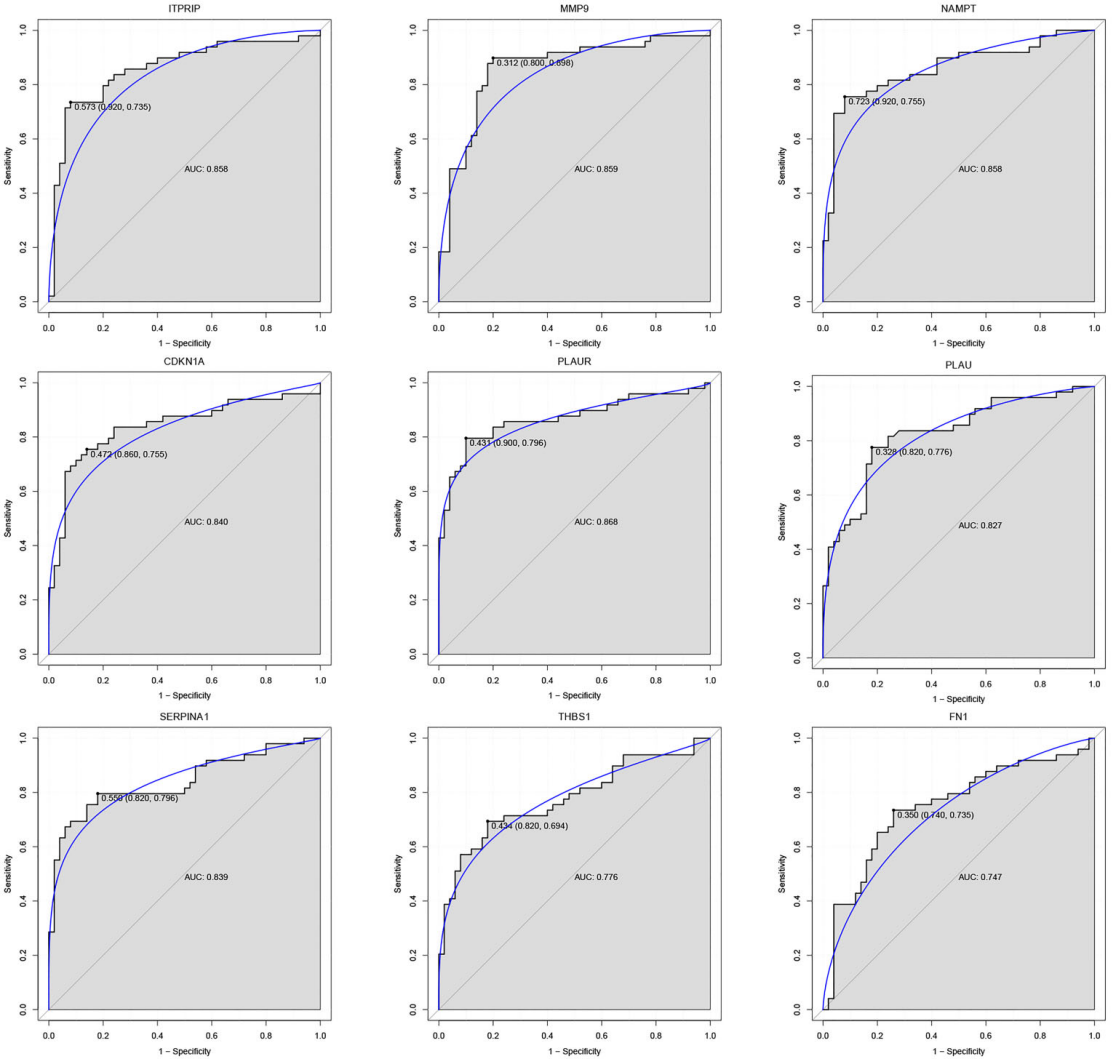
Receiver operating characteristic (ROC) curves of the 9 risk feature genes in the training dataset. AUC: area under the ROC curve.

**Figure 6:**
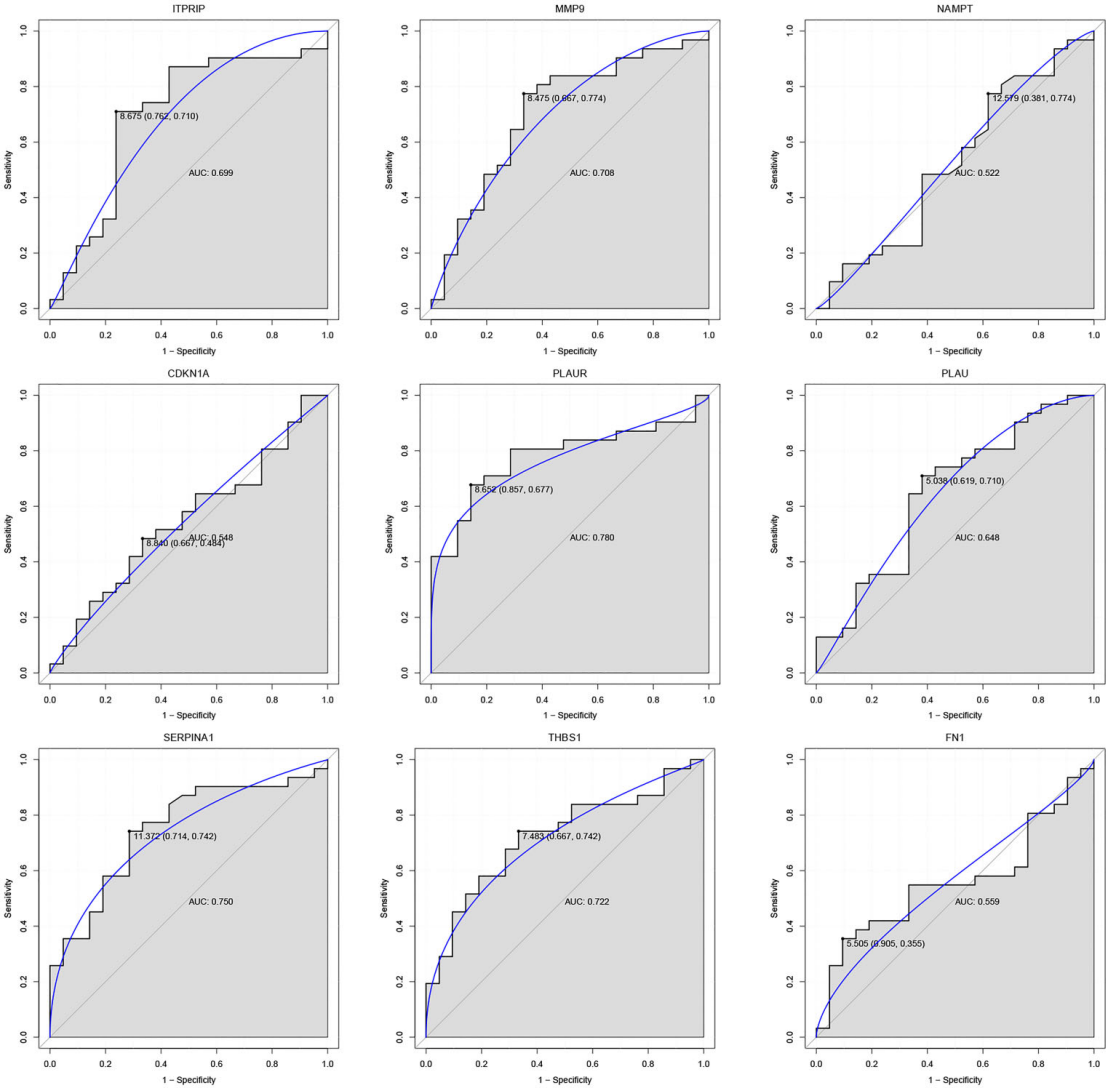
Receiver operating characteristic (ROC) curves of the 9 risk feature genes in the validation dataset. AUC: area under the ROC curve.

For the 9 risk feature genes selected, SVM was applied to construct a classification model, which was compared with RF, and it was found that both models had high AUC values (Supplementary Figure S2). In addition, we conducted ROC analysis and calculated AUC values for 9 biomarkers in both the training and validation datasets. All 9 genes showed AUC values >0.7 in the training dataset, indicating higher diagnostic accuracy (Figure 5). The AUC values of *MMP9*, *PLAUR*, *SERPINA1*, and *THBS1* in the validation dataset were also >0.7, suggesting that their diagnostic efficacy had a certain stability (Figure 6).

### Identification of anoikis-related clusters in AMI

**Figure 7:**
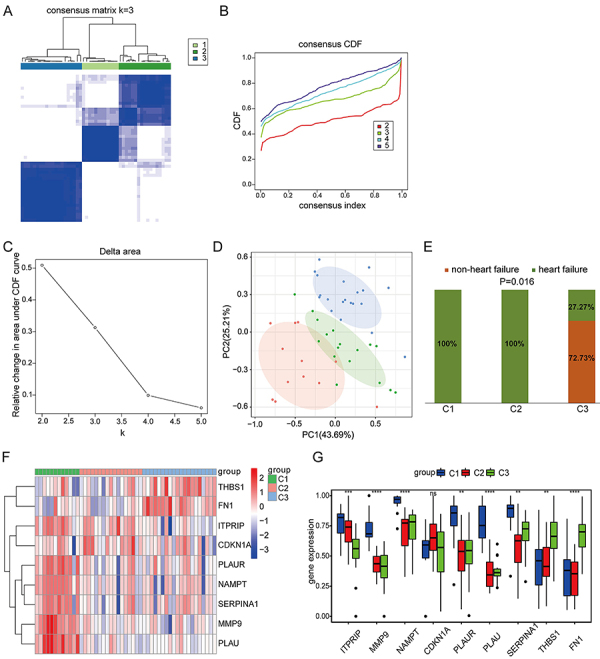
Consensus matrix analysis based on the expression of 9 risk feature genes in each acute myocardial infarction sample of the GSE66360 dataset. **A**, Consensus clustering matrix at k=2. **B**, Consensus index of CDF curves. **C**, CDF delta area curve. **D**, PCA of three clusters. **E**, Incidence of heart failure in three clusters based on the GSE59867 dataset. **F**, Heat map of the expression profiles of 9 risk feature genes in three clusters. **G**, Box plots of the expression of 9 risk feature genes in three clusters. Data are reported as median and interquartile range. **P<0.01, ***P<0.001, ****P<0.0001 (Kruskal-Wallis test). ns: non-significant. CDF: cumulative distribution function; PCA: principal component analysis.

To explore the different anoikis patterns of AMI, we performed consensus matrix analysis based on the expression of 9 risk feature genes in each AMI sample of the GSE66360 dataset. Our results showed that k=3 was an ideal choice, and each sample in the cluster showed significant correlation (Figure 7A-C). PCA indicated significant differences among the three clusters (Figure 7D). Furthermore, analysis of the GSE59867 dataset revealed that three clusters exhibited significant differences in heart failure incidence (P=0.016). Clusters 1 and 2 had a 100% incidence, markedly higher than cluster 3 at 27.27% (Figure 7E), suggesting that different clusters may predict differences in the risk of AMI patients progressing to heart failure. The heatmap displays the gene expression profiles of 9 risk feature genes in three clusters (Figure 7F). The gene expression results showed that *ITPRIP*, *MMP9*, *NAMPT*, *PLAUR*, *PLAU*, and *SERPINA1* were highly expressed in cluster 1, while the *THBS1* and *FN1* were highly expressed in cluster 3 (Figure 7G).

### Validation of the hub DEARGs by RT-qPCR

**Figure 8:**
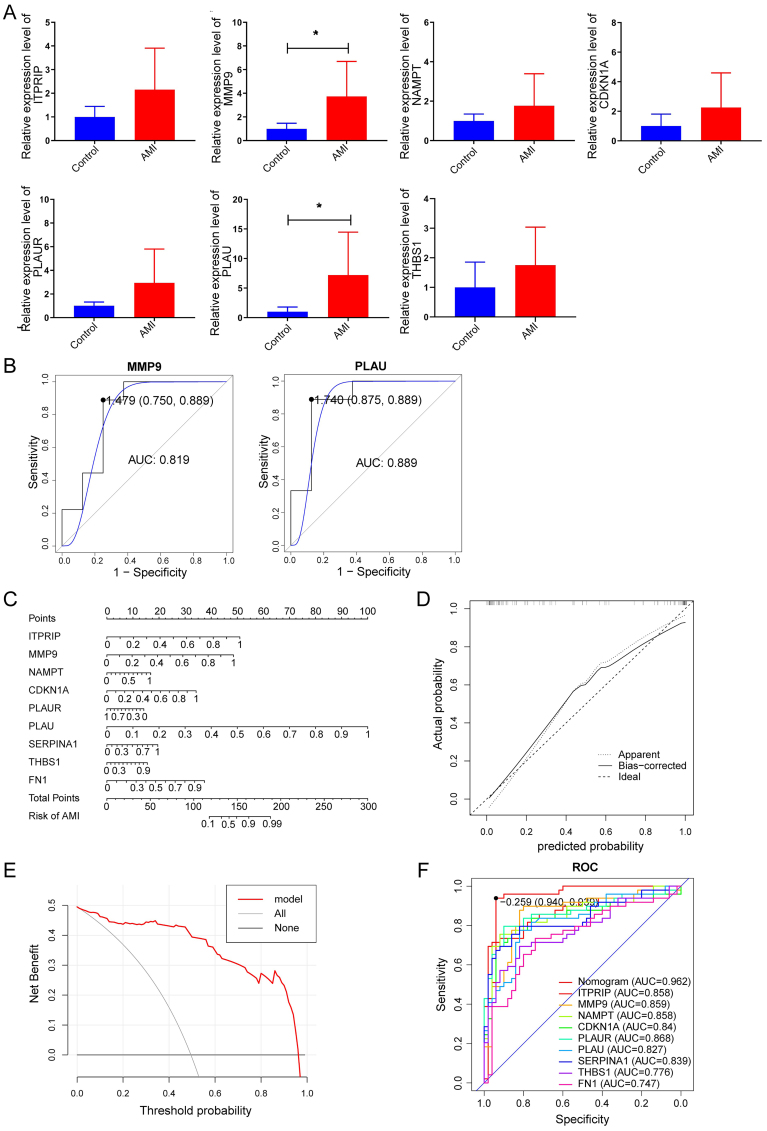
Establishment and verification of the nomogram. **A**, Gene expression was evaluated in blood samples from acute myocardial infarction (AMI) patients and healthy controls by RT-qPCR. Data are reported as mean and SD. *P<0.05 (*t*-test). **B**, Nomogram for the diagnosis of AMI. **C**, Calibration curve of the nomogram. **D**, Decision curve analysis of the nomogram. **E**, ROC curve: AUC of 0.962. **F**, ROC curves assessing the prognostic accuracy of nomogram and individual genes. ROC: receiver operating characteristic. AUC: area under the ROC curve.

To verify the reliability of bioinformatics data, we randomly selected 7 genes from the feature genes for RT-qPCR validation, including *ITPRIP*, *MMP9*, *NAMPT*, *CDKN1A*, *PLAUR*, *PLAU*, and *THBS1*. The RT-qPCR results demonstrated that the 7 genes were up-regulated in AMI patients (Figure 8A). It was noteworthy that the expression levels of *MMP9* and *PLAU* were markedly elevated in the AMI patients. The ROC curves further showed that *MMP9* and *PLAU* demonstrated a better performance in diagnosing AMI, with AUC values >0.8 (Figure 8B). These preliminary findings suggest that they hold potential as promising biomarkers for diagnosing AMI.

### Establishment and verification of the nomogram

A nomogram for the diagnosis of AMI was constructed by integrating the above 9 risk feature genes (Figure 8C). The accuracy of the nomogram was evaluated using calibration curve analysis, and the results showed that the calibration curve was close to the standard curve, with a good predictive ability (Figure 8D). DCA was used to evaluate the clinical value of the nomogram, and the curve indicates that AMI patients can benefit from the nomogram (Figure 8E). The AUC value of this nomogram was 0.962, which was higher than the AUC value of individual genes, suggesting a better predictive ability (Figure 8F).

## Discussion

AMI is associated with high morbidity and mortality, and early diagnosis and treatment can improve patients' survival rates (15). Unfortunately, the expression of common AMI biomarkers, including cTns, is also increased in other myocardial injuries (16). In recent years, considerable advances have been made in the regulatory mechanisms of AMI, with numerous promising diagnostic and therapeutic targets identified (17). Nevertheless, the clinical application of these findings still requires further validation. Meanwhile, the combination of multiple biomarkers may facilitate the early diagnosis and treatment of AMI.

Anoikis is a programmed cell death mechanism triggered by the loss of cell-ECM adhesion, serving as a critical biological defense against aberrant cell proliferation and migration (18). The ECM plays a significant role in cardiac remodeling and fibrosis in patients with AMI (9). In light of these findings, we postulated that anoikis may also be implicated in the potential mechanism of AMI. A total of 21 DEARGs were screened from peripheral blood samples of normal and AMI patients. The GO analysis showed that DEARGs were mainly enriched in the regulation of peptidase activity and ECM, indicating that DEARGs may be involved in the occurrence of AMI through these pathways. Peptidases are enzymes that cleave (poly) peptide bonds, regulating many important physiological processes such as blood coagulation, cell apoptosis, and non-specific protein turnover (19). Previous studies showed that elevated levels of an endopeptidase, matrix metalloproteinase (MMPs), were associated with the development of AMI (20). MMPs maintain vascular integrity by decomposing ECM and achieve vascular remodeling by promoting cell turnover, inflammatory signaling, angiogenesis, and collagen degradation (21). Moreover, the complement system was considered a major contributor to improving myocardial injury (22). Consistently, KEGG enrichment analysis of DEARGs showed that they are involved in complement and coagulation cascades. In conclusion, DEARGs may contribute to the onset and progression of AMI via these signaling pathways and may therefore represent potential diagnostic and therapeutic targets for AMI.

It has been demonstrated that immune cell recruitment and activation in AMI drive inflammatory cascades that paradoxically modulate both tissue damage and repair (23). Notably, dysregulated inflammatory responses have become a focus of therapeutic intervention, with IL-34-centered immunomodulation showing promise in preventing heart failure after AMI (24). Skorska et al. (25) found that CD4+ T cells play a major protective role during myocardial infarction. Mast cells have obvious inflammatory characteristics and regulate the metabolism of fibrous tissue in the process of cardiac tissue remodeling (26). Macrophages play a role in clearing necrotic tissue and secreting pro-decomposition mediators during inflammation resolution (27). Our research also found differences in the infiltration levels of some immune cells between the AMI and control groups, such as T cells CD4 memory resting, mast cells, and macrophages, indicating the important regulatory role of immune cells in AMI. Furthermore, the correlation analysis between DEARGs and immune cells revealed that aberrant expression of some DEARGs may be involved in AMI pathology by affecting immune cell infiltration. It was reported that in the first few hours of AMI, neutrophils are recruited into the infarcted myocardium, causing local inflammation (28). This leads to ECM excessive degradation by overexpressing MMP9, thereby disrupting cardiac tissue remodeling (29). In AMI, monocytes are recruited to the ischemic myocardium via adhesion molecules, promoting the vascular inflammatory response (30). This process may be regulated by PLAU (urokinase-type plasminogen activator, namely uPA), which drives the vascular inflammatory cascade by regulating monocyte adhesion to the vascular endothelium (31). RT-qPCR validation in the present study revealed that the expression levels of *MMP9* and *PLAU* were significantly increased in AMI, further confirming that their abnormal expression might have a potential impact on the AMI progression. Although there is a lack of direct evidence that immune cell alterations are involved in AMI through *MMP9*/*PLAU* regulation and anoikis mediation, our study confirmed that there was a certain association between them.

Machine learning (ML) uses data and algorithms to mimic the way humans learn and solve problems, which has been widely applied in risk classification for AMI (32). This study screened 9 feature genes through LASSO, SVM, and RF, including *ITPRIP*, *MMP*-9, *NAMPT*, *CDKN1A*, *PLAUR*, *PLAU*, *SERPINA1*, THBS1, and *FN1*. These genes have shown good predictive ability in the prediction of AMI, with all AUCs >0.7. Previous studies have shown that these genes are directly or indirectly associated with myocardial injury. *ITPRIP* induced the accumulation of mitochondrial calcium and reactive oxygen species through the ITPR2 channel, which was related to a decrease in cardiac contractility (33). It is well known that AMI leads to a dramatic decrease in the contractility of the infarcted myocardium, suggesting that *ITPRIP* may play an important role in myocardial dysfunction after AMI. MMP-9 increases during AMI, and its serum levels are associated with acute cardiomyopathy (34). The potential biomarker of AMI, CDR1as, promotes arrhythmia in myocardial infarction by directly targeting the NAMPT and causing mitochondrial dysfunction in AMI (35). The deacetylation of P21 protein encoded by *CDKN1A* plays a crucial role in myocardial cell proliferation and may be a new therapeutic strategy for myocardial infarction (36). *PLAU* and *PLAUR* encode uPA and its receptor (uPAR), and functional variants in these genes may influence susceptibility to myocardial infarction (37). *SERPINA1* is the most abundant serine protease inhibitor, and its genetic deficiency is associated with increased cardiovascular risk (38). *THBS1* positively regulates vascular smooth muscle cell proliferation and is believed to be associated with the pathogenesis of AMI (39). *FN1* encodes fibronectin, an essential component of the ECM, and *FN1* knockdown under hypoxic conditions reverses *HOTAIR* overexpression-mediated effects on H9c2 cell (an *in vitro* AMI model) viability and apoptosis (40). Although there are no mechanistic studies on the involvement of these genes in AMI through anoikis, previous evidence and our research suggest that these 9 risk feature genes can serve as targets for the diagnosis of AMI.

Consistent cluster analysis identified three AMI clusters with significant differences in heart failure incidence. Clusters 1 and 2 AMI patients, with a 100% heart failure incidence, should be closely monitored and prioritized for early intervention. Cluster 3 patients, with a lower incidence of 27.27%, may require less aggressive monitoring. These clusters could predict and stratify heart failure risk in AMI patients. This was consistent with previous evidence that anoikis is associated with adverse cardiac remodeling (18), reinforcing the clinical value of identifying clusters. Notably, the expression levels of the 9 risk feature genes showed significant differences among different clusters, and the associations between these genes and different immune cells may indirectly reflect the immune status of the myocardial microenvironment. Future studies should validate the regulatory effects of these genes on immune cell function by *in vitro* experiments to explore cluster-based hierarchical immunomodulatory strategies. In addition, we constructed a nomogram for AMI diagnosis based on the expression of 9 risk feature genes. The expression of each gene corresponds to a score, and the sum of the scores of all genes can provide the risk of AMI. The nomogram exhibited good reliability and validity, and can be used to diagnose AMI.

Despite the identification of 9 risk feature genes associated with AMI and the construction of a nomogram for AMI diagnosis, the study still has some limitations. Firstly, the data source of this study was derived from the GEO public database, which may be subject to potential biases in dataset selection. Secondly, the small sample size used for RT-qPCR verification, with only two genes showing significant differences, may weaken the translational significance of the results. Therefore, it is necessary to include large-scale clinical samples to further confirm these preliminary findings. Thirdly, the retrospective data prevented the analysis of comorbidity effects on gene expression and the integration of clinical parameters into the diagnostic model. Although the matched baseline characteristics in the RT-qPCR validation cohort suggested minimal comorbidity confounding, future studies should validate marker specificity through comorbidity-stratified analysis and incorporate comprehensive clinical data to improve model translatability. Fourthly, although our RT-qPCR validation was randomly conducted on 7 genes and had limited statistical validity, and main conclusions remain dependent on bioinformatics analysis, it provides preliminary support for confirming the significance of these genes. Further validation through protein expression assays (such as Western blot) and functional experiments (gene knockout/overexpression) is essential to enhance the reliability and clinical applicability of the results. Finally, our study investigated the correlation between immune infiltration and DEARGs, which may offer a theoretical foundation for the immunotherapy of AMI. Nevertheless, the immunotherapy regimen for AMI necessitates meticulous deliberation and formulation, in addition to further *in vivo* and *in vitro* research validation.

In summary, this study comprehensively analyzed the differential expression of ARGs and immune profiles in normal and AMI peripheral blood samples, screened 9 risk feature genes that can be used to predict AMI, and constructed an AMI diagnostic model with strong predictive performance. The study of differential expression patterns of ARGs in AMI not only enhances our comprehension of the refractory nature of the disease but also facilitates the advancement of personalized and precise immunotherapy strategies. This study provides a novel perspective for the early diagnosis and therapeutic improvements in patients with AMI, which requires larger datasets or combining bioinformatics with experimental studies for validation in the future.

## Supplementary Materials

Supplementary Material Click to view [pdf].

## Data Availability

All data generated or analyzed during this study are included in this published article.

## References

[1] Gao J, Zhang X, Xu M, Deng S, Chen X (2023). The efficacy and safety of sacubitril/valsartan compared with ACEI/ARB in the treatment of heart failure following acute myocardial infarction: a systematic review and meta-analysis of randomized controlled trials. Front Pharmacol.

[2] Vanherle L, Lidington D, Uhl FE, Steiner S, Vassallo S, Skoug C (2022). Restoring myocardial infarction-induced long-term memory impairment by targeting the cystic fibrosis transmembrane regulator. EbioMedicine.

[3] Demeulenaere O, Mateo P, Ferrera R, Chiaroni PM, Bizé A, Dai J (2023). Assessment of coronary microcirculation alterations in a porcine model of no-reflow using ultrasound localization microscopy: a proof of concept study. EbioMedicine.

[4] Hayward CJ, Batty JA, Westhead DR, Johnson O, Gale CP, Wu J (2023). Disease trajectories following myocardial infarction: insights from process mining of 145 million hospitalisation episodes. EbioMedicine.

[5] Chen Y, Tao Y, Zhang L, Xu W, Zhou X (2019). Diagnostic and prognostic value of biomarkers in acute myocardial infarction. Postgrad Med J.

[6] Shi HT, Huang ZH, Xu TZ, Sun AJ, Ge JB (2022). New diagnostic and therapeutic strategies for myocardial infarction via nanomaterials. EbioMedicine.

[7] Taddei ML, Giannoni E, Fiaschi T, Chiarugi P (2012). Anoikis: an emerging hallmark in health and diseases. J Pathol.

[8] Chiarugi P, Giannoni E (2008). Anoikis: a necessary death program for anchorage-dependent cells. Biochem Pharmacol.

[9] Contessotto P, Spelat R, Ferro F, Vysockas V, Krivickienė A, Jin C (2023). Reproducing extracellular matrix adverse remodelling of non-ST myocardial infarction in a large animal model. Nat Commun.

[10] Traverse JH (2012). Using biomaterials to improve the efficacy of cell therapy following acute myocardial infarction. J Cardiovasc Transl Res.

[11] Badia-Ramentol J, Linares J, Gómez-Llonin A, Calon A (2021). Minimal residual disease, metastasis and immunity. Biomolecules.

[12] Ong SB, Hernández-Reséndiz S, Crespo-Avilan GE, Mukhametshina RT, Kwek XY, Cabrera-Fuentes HA (2018). Inflammation following acute myocardial infarction: multiple players, dynamic roles, and novel therapeutic opportunities. Pharmacol Ther.

[13] Westman PC, Lipinski MJ, Luger D, Waksman R, Bonow RO, Wu E (2016). Inflammation as a driver of adverse left ventricular remodeling after acute myocardial infarction. J Am Coll Cardiol.

[14] Ibanez B, James S, Agewall S, Antunes MJ, Bucciarelli-Ducci C, Bueno H (2018). 2017 ESC Guidelines for the management of acute myocardial infarction in patients presenting with ST-segment elevation: The Task Force for the management of acute myocardial infarction in patients presenting with ST-segment elevation of the European Society of Cardiology (ESC). Eur Heart J.

[15] Bala M, Catena F, Kashuk J, De Simone B, Gomes CA, Weber D (2022). Acute mesenteric ischemia: updated guidelines of the World Society of Emergency Surgery. World J Emerg Surg.

[16] Katrukha IA, Katrukha AG (2021). Myocardial Injury and the release of troponins I and T in the blood of patients. Clin Chem.

[17] Lan C, Chen C, Qu S, Cao N, Luo H, Yu C (2022). Inhibition of DYRK1A, via histone modification, promotes cardiomyocyte cell cycle activation and cardiac repair after myocardial infarction. EbioMedicine.

[18] Michel JB (2003). Anoikis in the cardiovascular system: known and unknown extracellular mediators. Arterioscler Thromb Vasc Biol.

[19] Obaha A, Novinec M (2023). Regulation of peptidase activity beyond the active site in human health and disease. Int J Mol Sci.

[20] Owolabi US, Amraotkar AR, Coulter AR, Singam NSV, Aladili BN, Singh A (2020). Change in matrix metalloproteinase 2, 3, and 9 levels at the time of and after acute atherothrombotic myocardial infarction. J Thromb Thrombolysis.

[21] Bonnans C, Chou J, Werb Z (2014). Remodelling the extracellular matrix in development and disease. Nat Rev Mol Cell Biol.

[22] Panagiotou A, Trendelenburg M, Osthoff M (2018). The lectin pathway of complement in myocardial ischemia/reperfusion injury-review of its significance and the potential impact of therapeutic interference by C1 esterase inhibitor. Front Immunol.

[23] Huang S, Frangogiannis NG (2018). Anti-inflammatory therapies in myocardial infarction: failures, hopes and challenges. Br J Pharmacol.

[24] Zhuang L, Zong X, Yang Q, Fan Q, Tao R (2023). Interleukin-34-NF-κB signaling aggravates myocardial ischemic/reperfusion injury by facilitating macrophage recruitment and polarization. EbioMedicine.

[25] Skorska A, von Haehling S, Ludwig M, Lux CA, Gaebel R, Kleiner G (2015). The CD4(+) AT2R(+) T cell subpopulation improves post-infarction remodelling and restores cardiac function. J Cell Mol Med.

[26] Legere SA, Haidl ID, Légaré JF, Marshall JS (2019). Mast cells in cardiac fibrosis: new insights suggest opportunities for intervention. Front Immunol.

[27] Tourki B, Halade G (2017). Leukocyte diversity in resolving and nonresolving mechanisms of cardiac remodeling. FASEB J.

[28] Ma Y, Yabluchanskiy A, Lindsey ML (2013). Neutrophil roles in left ventricular remodeling following myocardial infarction. Fibrogenesis Tissue Repair.

[29] Tao ZY, Cavasin MA, Yang F, Liu YH, Yang XP (2004). Temporal changes in matrix metalloproteinase expression and inflammatory response associated with cardiac rupture after myocardial infarction in mice. Life Sci.

[30] Sager HB, Dutta P, Dahlman JE, Hulsmans M, Courties G, Sun Y (2016). RNAi targeting multiple cell adhesion molecules reduces immune cell recruitment and vascular inflammation after myocardial infarction. Sci Transl Med.

[31] Zhao R, Ren S, Moghadasain MH, Rempel JD, Shen GX (2014). Involvement of fibrinolytic regulators in adhesion of monocytes to vascular endothelial cells induced by glycated LDL and to aorta from diabetic mice. J Leukoc Biol.

[32] Al-Zaiti SS, Martin-Gill C, Zàgre-Hemsey JK, Bouzid Z, Faramand Z, Alrawashdeh MO (2023). Machine learning for ECG diagnosis and risk stratification of occlusion myocardial infarction. Nat Med.

[33] Saemann L, Wächter K, Georgevici AI, Pohl S, Hoorn F, Veres G (2024). Transcriptomic changes in the myocardium and coronary artery of donation after circulatory death hearts following *ex vivo* machine perfusion. Int J Mol Sci.

[34] El-Aziz TAA, Mohamed RH (2017). Matrix metalloproteinase-9 polymorphism and outcome after acute myocardial infarction. Int J Cardiol.

[35] Liu Y, Wang J, Zhao X, Li W, Liu Y, Li X (2023). CDR1as promotes arrhythmias in myocardial infarction via targeting the NAMPT-NAD(+) pathway. Biomed Pharmacother.

[36] Li B, Li M, Li X, Li H, Lai Y, Huang S (2019). Sirt1-inducible deacetylation of p21 promotes cardiomyocyte proliferation. Aging (Albany NY).

[37] Xu J, Li W, Bao X, Ding H, Chen J, Zhang W (2010). Association of putative functional variants in the PLAU gene and the PLAUR gene with myocardial infarction. Clin Sci (Lond).

[38] Curjuric I, Imboden M, Bettschart R, Caviezel S, Dratva J, Pons M (2018). Alpha-1 antitrypsin deficiency: From the lung to the heart?. Atherosclerosis.

[39] Wang Y, Xian H (2022). Identifying genes related to acute myocardial infarction based on network control capability. Genes (Basel).

[40] Yao J, Ma R, Wang C, Zhao G (2022). LncRNA-HOTAIR Inhibits H9c2 apoptosis after acute myocardial infarction via miR-206/FN1 axis. Biochem Genet.

