# Supplementary material for: Identification of anoikis-related biomarkers linking immune infiltration to acute myocardial infarction
Source: Braz J Med Biol Res. 2026 Jan 9;58:e14989. doi: 10.1590/1414-431X2025e14989 (PMC12801198; doi:10.1590/1414-431X2025e14989)
Supplement: Supplementary Material [file 1414-431X-bjmbr-58-e14989-suppl.pdf]

Figure S1. Comparison of gene expression distribution before (**A**) and after (**B**) data normalization.

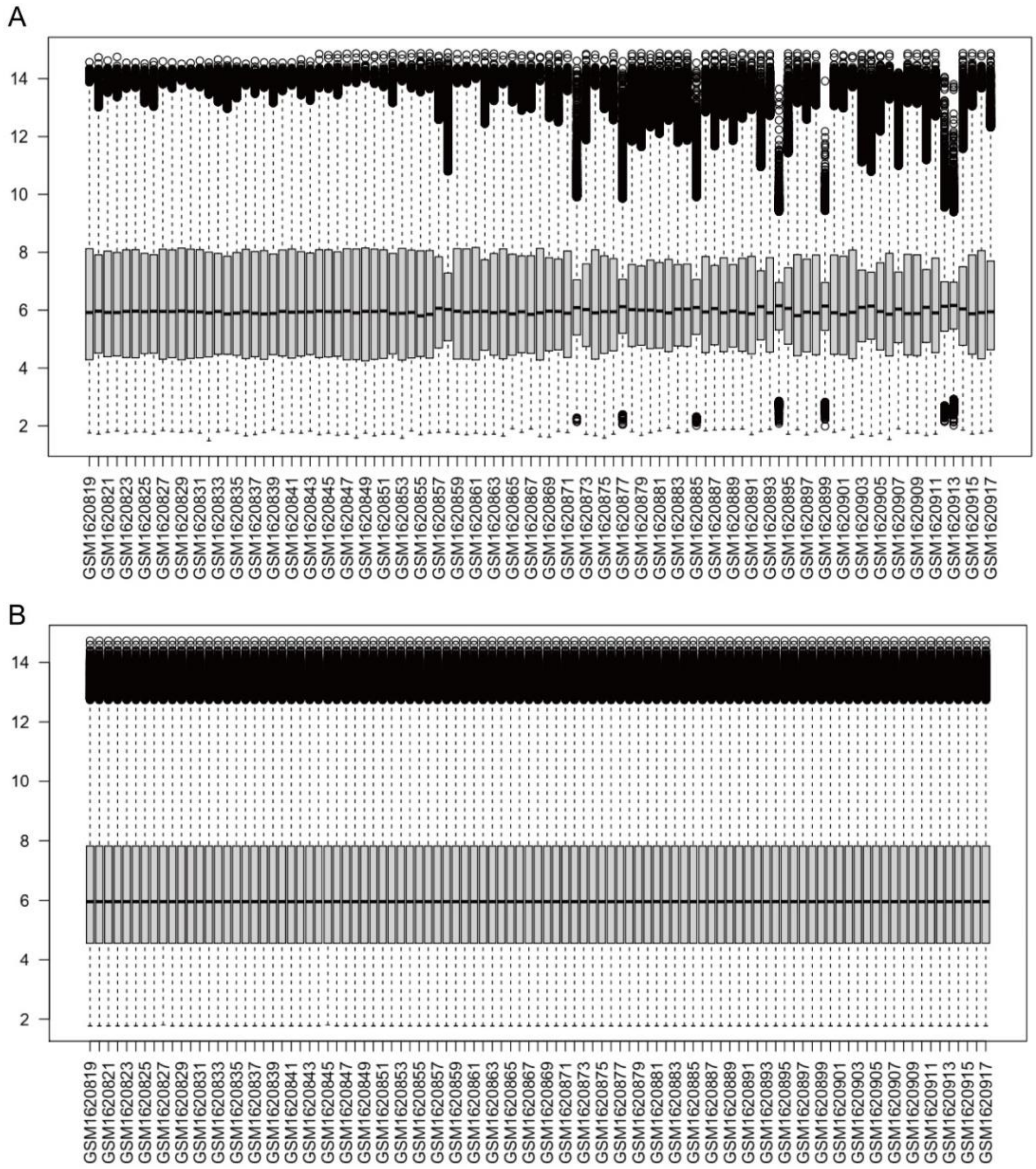

Figure S2. Validation of support vector machine (SVM) and random forest (RF) models. A, ROC curve of SVM model in the training dataset. B, ROC curve of RF model in the training dataset. C, ROC curve of SVM model in the validation dataset. D, ROC curve of RF model in the validation dataset. ROC: receiver operating characteristic; AUC: area under the ROC curve.

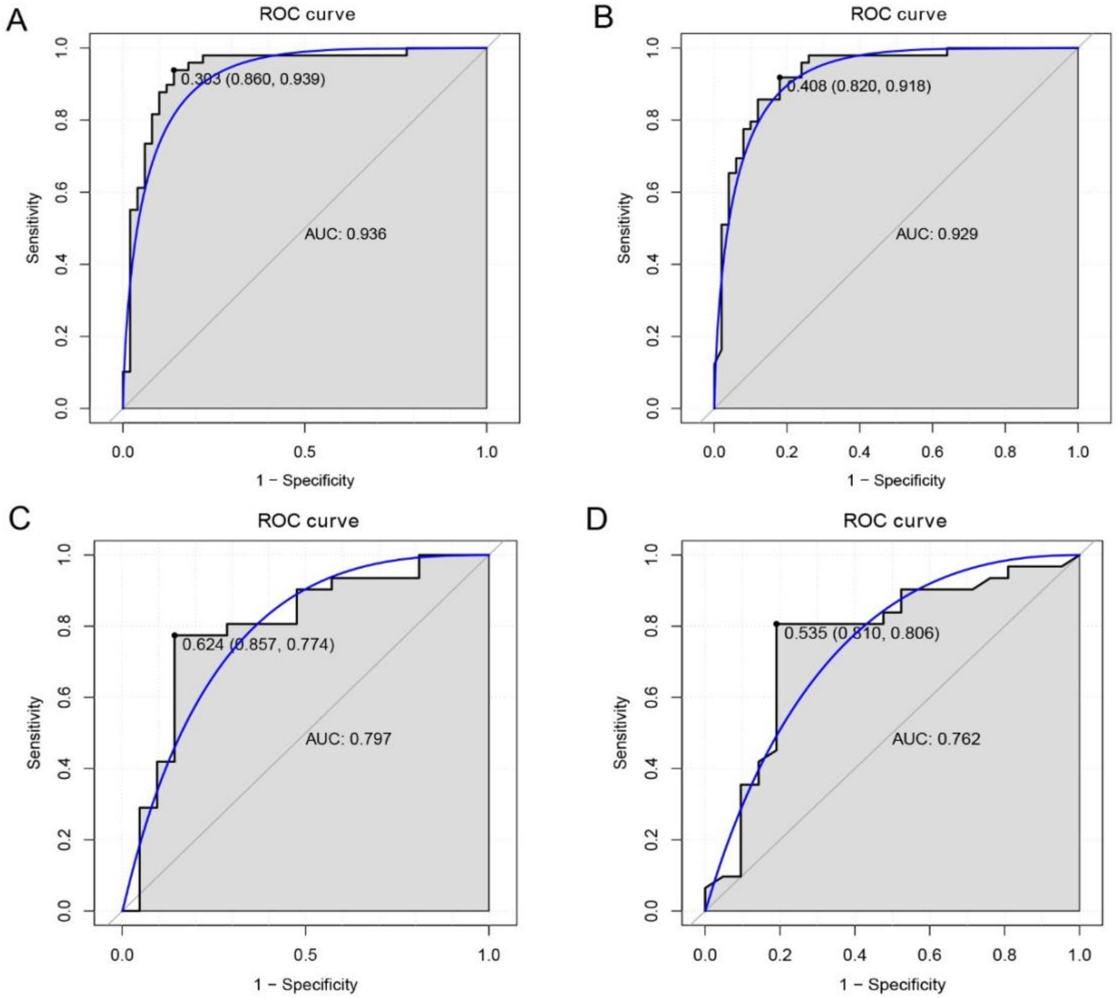

Table S1. Clinical information of 17 subjects of acute myocardial infarction (AMI) patients and controls.

Characteristics	Control (n=9)	AMI (n=8)	P value
Age (years)	62.67 (13.05)	64.00 (16.51)	0.855
Gender (Male, %)	6 (66.7)	6 (75.0)	1
Height (m)	1.68 (0.12)	1.63 (0.06)	0.296
Weight (kg)	67.89 (10.94)	66.25 (18.66)	0.826
Body mass index (kg/m²)	24.12 (4.06)	24.64 (5.95)	0.835
History of hypertension (N, %)	8 (88.9)	5 (62.5)	0.479
History of diabetes (N, %)	9 (100.0)	8 (100.0)	NA
History of smoking (N, %)	9 (100.0)	5 (62.5)	0.165
History of drinking (N, %)	9 (100.0)	6 (75.0)	0.399
History of acute myocardial infarction (N, %)	9 (100.0)	8 (100.0)	NA
History of atherosclerosis (N, %)	9 (100.0)	8 (100.0)	NA
Low-density lipoprotein (mmol/L)	2.98 (0.54)	3.03 (0.97)	0.906
High-density lipoprotein (mmol/L)	1.26 (0.26)	1.16 (0.32)	0.491
Total cholesterol (mmol/L)	3.87 (0.59)	4.97 (1.37)	0.048
Triglyceride (mmol/L)	2.19 (0.98)	1.46 (1.13)	0.191

Unless otherwise specified, data are reported as means±SD. Student’s *t*-test or chi-squared test.

Table S2. Primer sequence for quantitative real-time PCR.

Genes	Sequence (5'–3')
GAPDH-F	GGAGCGAGATCCCTCCAAAAT
GAPDH-R	GGCTGTTGTCATACTTCTCATGG
ACTB-F	CATGTACGTTGCTATCCAGGC
ACTB-R	CTCCTTAATGTCACGCACGAT
ITPRIP-F	ATGACTCGGACCTGTACTTTGT
ITPRIP-R	CCTGAGGAAGTGTCGCTCATAG
MMP9-F	GGGACGCAGACATCGTCATC
MMP9-R	TCGTCATCGTCGAAATGGGC
NAMPT-F	GCTTTCCCACTACTCCAGCC
NAMPT-R	TTTAGCCTCCTCCCTTCCCT
CDKN1A-F	TGTCCGTCAGAACCCATGC
CDKN1A-R	AAAGTCGAAGTTCCATCGCTC
PLAUR-F	TGTAAGACCAACGGGGATTGC
PLAUR-R	AGCCAGTCCGATAGCTCAGG
THBS1-F	TGCTATCACAACGGAGTTCAGT
THBS1-R	GCAGGACACCTTTTTGCAGATG
